# The impact of supervised physical exercise on chemokines and cytokines in recovered COVID-19 patients

**DOI:** 10.3389/fimmu.2022.1051059

**Published:** 2023-01-04

**Authors:** Tayrine Ordonio Filgueira, Paulo Roberto Cavalcanti Carvalho, Matheus Santos de Sousa Fernandes, Angela Castoldi, Ana Maria Teixeira, Renata Bezerra de Albuquerque, José Luiz de Lima-Filho, Fabrício Oliveira Souto

**Affiliations:** ^1^ Postgraduate Program in Biology Applied to Health, Center of Biosciences, Federal University of Pernambuco, Recife, Brazil; ^2^ Postgraduate Program in Surgery, Center for Medical Sciences, Federal University of Pernambuco, Recife, Brazil; ^3^ Postgraduate Program in Neuropsychiatry and Behavioral Sciences, Center of Medical Sciences, Federal University of Pernambuco, Recife, Brazil; ^4^ Keizo Asami Institute, Federal University of Pernambuco, Recife, Brazil; ^5^ Life Sciences Center, Agreste Academic Center, Federal University of Pernambuco, Caruaru, Brazil; ^6^ Faculty of Sport Sciences and Physical Education, Research Center for Sport and Physical Activity, University of Coimbra, Coimbra, Portugal

**Keywords:** supervised exercise protocol, home-based exercise, immune biomarkers, chemokines, cytokines, COVID-19

## Abstract

COVID-19 is an infectious disease caused by the SARS-CoV-2 virus, which induces a high release of pro-inflammatory chemokines and cytokines, leading to severe systemic disorders. Further, evidence has shown that recovered COVID-19 patients still have some symptoms and disorders from COVID-19. Physical exercise can have many health benefits. It is known to be a potent regulator of the immune system, which includes frequency, intensity, duration, and supervised by a professional. Given the confinement and social isolation or hospitalization of COVID-19 patients, the population became sedentary or opted for physical exercise at home, assuming the guarantee of the beneficial effects of physical exercise and reducing exposure to SARS-CoV-2. This study aimed to investigate the effects of a supervised exercise protocol and a home-based unsupervised exercise protocol on chemokine and cytokine serum levels in recovered COVID-19 patients. This study was a prospective, parallel, two-arm clinical trial. Twenty-four patients who had moderate to severe COVID-19 concluded the intervention protocols of this study. Participants were submitted to either supervised exercise protocol at the Clinical Hospital of the Federal University of Pernambuco or home-based unsupervised exercise for 12 weeks. We analyzed serum levels of chemokines (CXCL8/IL-8, CCL5/RANTES, CXCL9/MIG, CCL2/MCP-1, and CXCL10/IP-10) and cytokines (IL-2, IL-4, IL-6, IL-10, IL-17A, TNF-α, and IFN-γ). Before the interventions, no significant differences were observed in the serum levels of chemokines and cytokines between the supervised and home-based unsupervised exercise groups. The CXCL8/IL-8 (*p* = 0.04), CCL2/MCP-1 (*p* = 0.03), and IFN-γ (*p* = 0.004) levels decreased after 12 weeks of supervised exercise. In parallel, an increase in IL-2 (*p* = 0.02), IL-6 (*p* = 0.03), IL-4 (*p* = 0.006), and IL-10 (*p* = 0.04) was observed after the supervised protocol compared to pre-intervention levels. No significant differences in all the chemokines and cytokines were found after 12 weeks of the home-based unsupervised exercise protocol. Given the results, the present study observed that supervised exercise was able to modulate the immune response in individuals with post-COVID-19, suggesting that supervised exercise can mitigate the inflammatory process associated with COVID-19 and its disorders.

**Clinical trial registration:**

https://ensaiosclinicos.gov.br/rg/RBR-7z3kxjk, identifier U1111-1272-4730.

## Introduction

Coronavirus disease 2019 (COVID-19) is a severe acute respiratory syndrome caused by the SARS-CoV-2 virus, spread worldwide and has persisted due to emerging variants (1). The main symptoms are fever, cough, tiredness, headache, chronic inflammation, diarrhea, loss of taste or smell, and skin irritation or discoloration of the fingers or toes (2). In the most severe form, the patients have respiratory difficulty, chest pain, motor capacity loss, hematological diseases, chronic obstructive pulmonary disease, chronic kidney disease, and immunosuppression (3). In addition, recent clinical trials have noticed that recovered COVID-19 patients still have some symptoms and disorders from COVID-19, such as fatigue (4), breathlessness, functional lung abnormalities (5), myocardial injury (6), abnormal inflammatory indicators (7) in different recovery stages. It indicates that continuous awareness of recovery patients should be done to assist in effective rehabilitation since the COVID-19 sequelae have been an international concern of public health.

SARS-CoV-2 acute infection is followed by overproduction and secretion of inflammatory cytokines, such as Interleukin (IL)-1beta, IL-2, IL-2R, IL-6, tumor necrosis factor (TNF) alpha, interferon-γ (IFN-γ), but also high levels of some anti-inflammatory cytokines as IL-4 and IL-10 (8). Meanwhile, monocytes, macrophages, and cells of the adaptive immune system, such as CD8 T and CD4 T cells, are mobilized to the respiratory tract and contribute to the secretion of IFN-γ, promoting additional inflammation and establishment of continued pro-inflammatory response (9). The inflammatory alterations are called “*cytokine release syndrome*” (CRS) and are associated with different clinical characteristics of COVID-19. Also, they have been correlated with the progression of the acute phase of the SARS-CoV-2 infection (9, 10). Finally, the high peripheral levels of IL-6 and IL-10 have been highlighted as a predictor of COVID-19 severity (11). That scenario must culminate in acute respiratory distress syndrome, multiple organ failure, and mortality from COVID-19 (12). In the more advanced stage of the disease, the immune response is impaired partly due to the NK, and CD8 T cells depletion, resulting in a consequent decrease in the secretion of IFN-γ, IL-2, and granzyme B from NK and CD8 T cells (13, 14).

Additionally, studies have shown that SARS-CoV-2 infection promotes a hyperinflammatory state within the lungs, building up to high systemic concentrations of several chemokines, and inflammatory lipid mediators (9, 15). Zaid et al. (2021) found an overproduction of 11 chemokines, mainly with CXCL1 and CXCL8 being 200-fold more abundant than IL-6 and TNF-α in the Bronchoalveolar lavage fluid of patients with severe COVID-19 (16). The expression of hypoxia-inducible factor 1α and its inflammation-related transcription targets, including CXCL8, CXCR1, CXCR2, and CXCR4 was observed in bronchoalveolar cells (17). Different studies have found high serum levels of the chemokines CXCL8/IL-8, IP-10, GM-CSF, CCL2/MCP-1, CCL3/MIP-1α, CXCL10/IP-10, CCL5/RANTES, and CCL20/MIP-3α, in patients with moderate and severe forms of the COVID-19 (15, 18). It is shown the chemokine CCL4/MIP1-β is upregulated in the pulmonary SARS-CoV-2 infection (19). Moreover, the increased serum concentrations of CXCL10/IP-10 were associated with mortality (18). Therefore, these chemokines have been identified as potential biomarkers of COVID-19 infection severity.

Ending the acute phase of SARS-Cov-2 infection, most people discharged from the hospital have experienced some COVID-19 chronic marks, such as respiratory sequelae, fatigue, elevated rates of multi-organ dysfunction, and poor exercise tolerance (20). This scenario might be explained since studies have reported high levels of pro-inflammatory cytokines in discharged patients (21, 22). Zhang etal. (23) observed TNF-α remains elevated in moderate to severe COVID-19, while peripheral levels of IL-6 returned to comparable healthy levels (24). Willems et al. (2021) noted that people three months discharged from the hospital had high levels of pro-inflammatory cytokines, such as IL-6, IL-1ra, and IL-18 (25). Peluso et al. (2022) have noticed 45% of COVID-19 patients who recovered from 6-8 months still have high significative levels of IL-6 (26). However, the remaining pro-inflammatory environment has yet to be seen in all studies investigating recovered COVID-19 patients. For example, Zhou et al. (2020) have investigated the impacts of COVID-19 on recovered patients’ cognitive and immune functions. They did not observe any chronic effect in the plasma levels of interleukins IL-2, IL-4, IL-6, IL-10, TNF-α, IFN-γ, and PCR (27). Moreover, only a few clinical trials have investigated the impact of COVID-19 on chemokines peripheral levels after recovery. Gacci et al. (2021) have found pathological levels of IL-8 in the semen of 76.7% of investigated patients, which was associated with COVID-19 severity (28). While Manganotti et al. (2021) have noticed higher IL-8 levels in the serum of people who were four months recovered, which might be associated with peripheral nervous system disorder (29). Although great strong evidence is scarce, studies have strengthened the hypothesis that long-term inflammation is even years after recovery from COVID-19 (30).

Compelling evidence has pointed to exercise as a non-pharmacological therapeutic strategy to mitigate the consequences of COVID-19, including in the different stages of its recovery (31, 32). Many studies have shown that physical exercise stimulates the production and release of several myokines, which are associated with several benefits in various tissues, organs, and systems, including the immune system (33–35). These studies investigated physical exercise as a controlled exercise protocol with frequency, intensity, and duration, performed in an environment with infrastructures such as gyms and training centers. Muscle contraction induced by physical exercise produces and releases myokines, including IL-6 (36–38). In addition, the increase in serum IL-6 levels due to exercise is followed by increased anti-inflammatory cytokines such as IL-10 and IL-1ra (39). Then regular physical exercise might reduce the risk of a pathogenic inflammatory response such as the CRS, decreasing one’s risk of getting the severe form of COVID-19 (40). The controlled exercise protocol effectively modulates other important pathways of the inflammatory process present in this disease, which remain remnants of it, such as increased levels of chemokines. Bartlett et al. (2018) observed that supervised exercise was effective in promoting neutrophil migration through CXCL8/IL-8 (p=0.003) in patients with rheumatoid arthritis (41). Also, Middelbeek et al. (2021) showed that controlled moderate-intensity training increased MCP-1 concentrations in sedentary individuals (42).

During the high peaks of the pandemic, it was necessary to carry out confinement and social isolation. Social isolation proved to be, in fact, a barrier to the regular practice of physical activity by the population. After the confinement, the usual activities were not resumed, especially for people discharged from the COVID-19 pandemic. In this sense, home-based exercise was a viable alternative to promote the already evidenced benefits of physical exercise and avoid the damage of the sedentary lifestyle associated with COVID-19 (43). It is known that home-based exercise is an unsupervised, self-selected moderate-intensity exercise done at home based on an exercise booklet, videos, or apps. Evidence has elucidated the beneficial effect of home-based physical exercise, promoting greater adherence, increased physical and mental fitness, and improved fatigue, among others (43). Moreover, a few studies showed the effect of home-based exercise on the immune system of patients with various chronic diseases (44–46). Schwartz et al. (2021) showed an augment in the concentration of CD4+ and CD8+ T-cells producing IFN-γ due to 12 weeks of home-based exercise and nutritional advice in obese endometrial cancer survivors. These findings were associated with cardiovascular benefits (46). Also, Lee et al. (2013) have observed that 12 weeks of home-based uncontrolled exercise reduced TNF-α peripheral levels of colorectal cancer survivors (47). Although there is little evidence, it would appear logical to achieve the anti-inflammatory effects of supervised exercise that has already been applied to a large number of other chronic conditions with a home-based exercise protocol.

In this context, it is known that home-based exercise has been investigated in some chronic and inflammatory pulmonary diseases, including the COVID-19 scenario (48–50). Moreover, it was the physical exercise strategy most of the population chose when they faced the need for social isolation. Although there is good evidence about home-based exercise, several supervised physical exercise studies have widely supported physical exercise’s protective and curative effects in extensive contexts, including on the recovery from COVID-19 (32). Nevertheless, few studies evaluate the effect of supervision on a physical exercise strategy, and the implications on immune system-related outcomes, such as chemokines and cytokines in COVID-19 recovery. Since the coronavirus derivatives continue to spread globally, developing an effective therapy for its disorders may be necessary. Therefore, this study aims to investigate the effects of supervised exercise and home-based unsupervised exercise on chemokines and cytokines concentrations in the blood of recovered COVID-19 patients.

## Methods

### Trial design

This study was a prospective, two-arm, parallel, clinical trial. The groups were submitted to supervised exercise or home-based unsupervised exercise protocols. The study procedures were approved by the Research Ethics Board of The Federal University of Pernambuco (#4.492.660). This trial was pre-registered at The Brazilian Clinical Trials Registry (https://ensaiosclinicos.gov.br/rg/RBR-7z3kxjk).

### Participants

**Figure 1:**
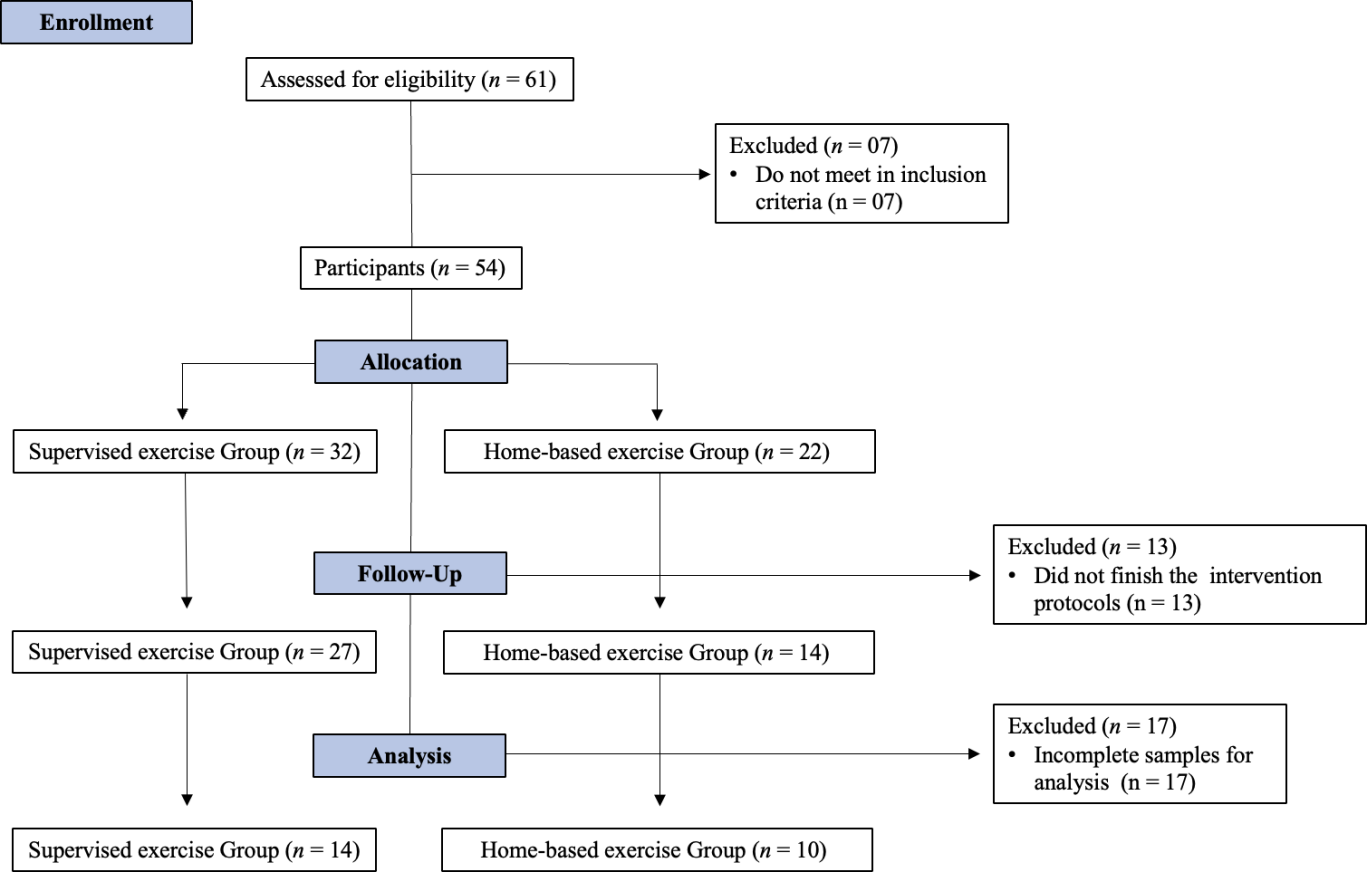
CONSORT flow diagram.

Fifty-four adults (between 18 to 60 years old) with moderate to severe COVID-19 were eligible to participate (**Figure 1**). All participants have taken the COVID-19 vaccine. Inclusion criteria were: (1) patients with 3 months of medical release from outpatient clinics and (2) who currently live in the metropolitan region of Recife, Pernambuco. Exclusion criteria included (1) patients who have limitations to performing the intervention protocols, (2) individuals who did not reach the cutoff points on the Mini-Mental State Examination (MMSE), and (3) who were hospitalized in the last 12 months before COVID-19 infection, and (4) individuals with decompensated heart failure and chronic obstructive pulmonary disease. A convenience sample size was used due to the emergency scenario of the COVID-19 pandemic.

### Study interventions

The supervised training protocol in the Clinical Hospital of the Federal University of Pernambuco was carried out in 12 weeks, totaling 36 sessions, and was performed three times a week on non-consecutive days. It was performed in a circuit form and used the following exercises: bench press, leg extension, low row, machine development, barbell curl, high articulated pull-up, triceps forehead, and abdominal, which were performed in the order described. A 40 seconds execution was performed in each exercise with an interval of 20 seconds between them, representing the time of equipment change, and sixty seconds of resting period after each round. Four rounds were performed in each training session, the first lap being performed with a light load to warm up. The machine exercise intensity was 60% of the maximum repetition value and 45 minutes duration (51, 52).

The home-based unsupervised group participated in the physical exercise at home, guided by a booklet, a reduced version of the Manual “Gymnastics to do at home” (53). for also 12 weeks, three times sessions/week on non-consecutive days. It was performed 10 min a warm-up followed by a circuit, and flexibility exercises of cool down. The muscular resistance home-based protocol was based on body weight for all muscle groups, three sets in each exercise, 8-10 repetitions, and sixty seconds of resting period between each set. It was chosen based on this reference as it had already been tried with individuals treated in public health services who had noncommunicable diseases (53). This is already evaluated material that proved viable and brought benefits to the target audience. Participants received the booklet and some instructions the week following the initial assessment. In addition, the participants of the home-based unsupervised group were followed by weekly telephone calls to monitor adherence to this exercise protocol.

### Measures

#### Analysis of chemokines and cytokines

Blood samples were collected in Vacutainer^®^ tubes without anticoagulant for the serum, five milliliters of blood were obtained per tube, and centrifuged at 1500 RPM for 10 min at 4°C. Two tubes were collected in the pre-stages and two in each post-intervention stage. The samples were used for the analysis of the chemokines CXCL8/IL-8, CCL5/RANTES, CXCL9/MIG, CCL2/MCP-1, and CXCL10/IP-10 and cytokines: IL-2, IL-4, IL-6, IL-10, TNF-α, IFN-γ, and IL-17-A, using chemokine and Th1/Th2/Th17 Cytometric Bead Array-CBA kits, respectively (BD Pharmingen). The reading of CBA was done by flow cytometer (BD Accuri™ C6). The analysis will be performed using the FCAP Array software (CA).

### Statistical analysis

Exploratory analysis and data adjustments were made to avoid data heterogeneity and the variability was performed in association with the normality test of the Shapiro-Wilk (n<50 participants). The group differences in baseline were assessed by the T-test. In addition, we conducted the Kruskal–Wallis test with multiple comparisons and the Dunn post-test, comparing the means of the different groups. Once age difference was observed, we applied a one-way analysis of covariance (ANCOVA) with age baseline values being a covariate. The significance value was set up at p<0.05 (5%). The prism V.9 GraphPad was used *(*GraphPad Software Inc, La Jolla, CA, USA). Furthermore, we utilized the Statistical Package for the Social Sciences (SPSS for Mac, version 20) to apply the ANCOVA test.

## Results

Initially, sixty-one individuals were contacted, and, based on the eligibility criteria, fifty-four adults aged 26-53 years were enrolled. Fifty-four individuals met all the eligibility criteria and participated in the twelve weeks of the training protocol. Thirteen participants withdrew from that study during the intervention period, since they did not conclude 36 sessions. At the end of the intervention, the samples of twenty-four participants were analyzed due to incomplete paired data (**Figure 1**). The group of participants who did supervise exercise protocol was named the gym group, also the group who did home-based unsupervised exercise protocol was named the home group. In the initial comparison, there were no significant differences between the gym and home groups in body mass, height, BMI, chemokines, and cytokine serum levels. These physical characteristics of the participants at the pre-intervention moment are shown in **Table 1**.

**Table 1:** Baseline characteristics of patients in both groups.

Items	Home	Gym	*P*
	Mean ± SD	Mean ± SD	
Age (years)	42.86 ± 10.81	57.30 ± 7.46	.00*
Body mass (kg)	84.72 ± 20.33	87.39 ± 15.30	0.73
Height (cm)	164.20 ± 11.50	164.10 ± 9.84	0.97
BMI (kg/m^2^)	31.84 ± 8.80	31.11 ± 4.79	0.98
IL-8 (pg/mL)	364.60 ± 273.70	367.10 ± 267.60	0.98
CXCL10 (pg/mL)	7966.00 ± 9788.00	2464.00 ± 2928.00	0.06
CCL5 (pg/mL)	110218.00 ± 10393.00	110835.00 ± 16256.00	0.91
CXCL9 (pg/mL)	2512.00 ± 2664.00	1169.00 ± 2504.00	0.22
CCL2 (pg/mL)	1300.00 ± 770.70	772.50 ± 437.00	0.10
IL-2 (pg/mL)	37.26 ± 4.05	39.81 ± 13.56	0.57
IL-4 (pg/mL)	106.50 ± 8.04	107.20 ± 13.80	0.90
IL-6 (pg/mL)	135.70 ± 38.10	239.10 ± 261.90	0.23
IL-10 (pg/mL)	76.80 ± 2.65	87.10 ± 17.80	0.08
TNF-α (pg/mL)	138.30 ± 24.80	166.80 ± 100.90	0.39
IFN-γ (pg/mL)	43.60 ± 15.70	38.70 ± 6.50	0.30
IL-17A (pg/mL)	275.40 ± 428.30	161.50 ± 224.40	0.40

SD, standard deviation; P, probability; kg, kilogra; BMI, Body Mass Index; IL, Interleukin; CXCL, chemokine (C-X-C motif) ligand; CCL, CC chemokine family; TNF, Tumor necrosis factor; IFN-γ, Interferon-gamma; and *, significant.

**Figure 2:**
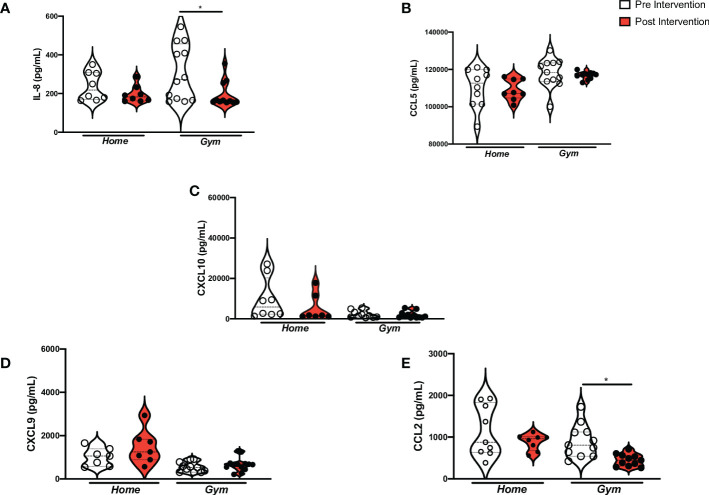
Chemokines levels in Supervised and home-based unsupervised exercise on recovered COVID-19 patients. ANOVA between Supervised and home-based unsupervised exercise on the chemokines from baseline to 12 weeks of intervention (Pre- and Post-intervention). Home, the home-based unsupervised exercise group; Gym, supervised exercise group; Serum levels; **(A)** IL-8: Interleukin- 8 (home (pre), n= 8, home (post), n= 8; gym (pre), n= 13, gym (post), n= 11; *p* = 0.04); **(B)** CCL5: CC chemokine family-5 (home (pre), n= 10, home (post), n= 8; gym (pre), n=11, gym (post), n=11; *p* = n.s.); **(C)** CXCL10, chemokine (C-X-C motif) ligand-10 (home (pre), n= 8, home (post), n= 7; gym (pre), n= 10, gym (post), n=11; *p* = n.s.); **(D)** CXCL9, chemokine (C-X-C motif) ligand-9 (home (pre), n= 8, home (post), n= 8; gym (pre), n= 11, gym (post), n= 13; *p* = n.s.); **(E)** CCL2, CC chemokine family-2 (home (pre), n= 10, home (post), n= 10; gym (pre), n= 13, gym (post), n= 12; *p* = 0.03); pg/mL, picogram per milliliter; *p< 0.05.

After the intervention, no changes were observed in the levels of chemokines in the home-based unsupervised exercise group compared to pre-intervention levels. However, serum levels of CXCL8/IL-8 (*p* = 0.04) and CCL-2/MCP-1 (*p* = 0.03) were decreased after supervised exercise in the gym group after twelve weeks (**Figure 2**). The IL-8 serum levels ranged in the gym as compared to the home group (Δ= -97.72 ± 205.9 pg/mL vs Δ= -154.8 ± 314.9 pg/mL). Moreover, CCL-2 also ranged in relationship to the home-based unsupervised group (Δ = -345.0 ± 357.5 pg/mL vs. Δ = -196.4 ± 749.5) (**Supplementary Figure 1**). There were no changes in serum levels of other chemokines (CCL-5, CXCL-9, and CXCL-10) in the gym or the home groups (**Figure 2**). In this sense, CCL-5 serum levels did not change significantly in both groups (Δ = 2229 ± 12895 pg/mL vs. Δ = -15217 ± 29922 pg/mL). Also, CXCL-9 did not range significantly between the gym and home groups (Δ = 165.0 ± 347.3 pg/mL vs. Δ = -383.8 ± 3078.0 pg/mL). Despite that, CXCL-10 did vary according to both exercise protocols (Δ = 254.7 ± 3465.0 pg/mL vs. Δ = -4195.0 ± 7743.0 pg/mL, *p* = 0.04) (**Supplementary Figure 1**).

**Figure 3:**
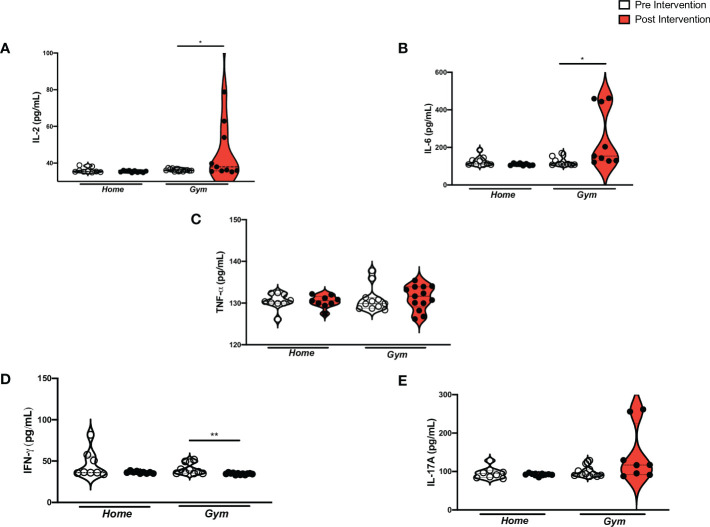
Pro-inflammatory cytokine levels in Supervised and home-based unsupervised exercise on recovered COVID-19 patients. ANOVA between Supervised and home-based unsupervised exercise on the pro-inflammatory cytokines from baseline to 12 weeks of intervention (Pre- and Post-intervention). Home, the home-based unsupervised exercise group; Gym, supervised exercise group; Serum levels; **(A)** IL-2: Interleukin-2 (home (pre), n= 9, home (post), n= 10; gym (pre), n= 13, gym (post), n= 12; *p* = 0.02); **(B)** IL-6: Interleukin-6 (home (pre), n= 9, home (post), n=8; gym (pre), n=10, gym (post), n=10; *p* = 0.03); **(C)** TNF-α: Tumor necrosis factor-alpha (home (pre), n= 9, home (post), n= 9; gym (pre), n= 12, gym (post), n= 14; *p* = n.s); **(D)** IFN-γ: Interferon-gamma (home (pre), n= 8, home (post), n= 9; gym (pre), n=11, gym (post), n=12; *p* = 0.004); **(E)** IL-17A, Interleukin-17A (home (pre), n= 8, home (post), n= 9; gym (pre), n= 12, gym (post), n= 8; *p* = n.s.); pg/mL, picogram per milliliter; *p<0.05 and **p≤ 0.01..

The cytokines IL-2 (*p* = 0.02) and IL-6 (*p* = 0.03) increased in individuals who performed 12 weeks of supervised physical exercise as compared to the baseline (**Figure 3**). The serum levels of IL-2 did range significantly when they were juxtaposed with the home-based unsupervised exercise (Δ = 12.18 ± 22.36 pg/mL vs Δ = -2.16 ± 4.34 pg/mL). Also, IL-6 peripheral levels ranged when the gym group was compared to the home group (Δ = 67.0 ± 195.1 pg/mL vs Δ = -17.18 ± 24.36 pg/mL) (**Supplementary Figure 2**). In parallel, the serum levels of IFN-γ (*p* = 0.004) decreased after supervised exercise in the gym group as compared to the baseline (**Figure 3**). When it was juxtaposed with the home group, they changed (Δ = -3.42 ± 7.46 pg/mL vs Δ = -8.06 ± 16.45 pg/mL) (**Supplementary Figure 2**). Despite that, there were no changes in TNF-α and IL-17A serum levels due to the supervised exercise protocol (**Figure 3**). Hence, TNF-α serum levels did not change significantly in both groups (Δ = -1.2 ± 6.14 pg/mL vs. Δ = 0.26 ± 2.30 pg/mL) (**Supplementary Figure 2**). Nonetheless, the difference in baseline age values between the groups significantly affected the variation in TNF-α serum levels in the gym group (*p* = 0.00) (**Supplementary Table 1**). Also, IL-17A did range significantly between the gym and home groups (Δ = 456.2 ± 748.7 pg/mL vs. Δ = -182.6 ± 427.2 pg/mL, *p* = 0.01) (**Supplementary Figure 2**).

**Figure 4:**
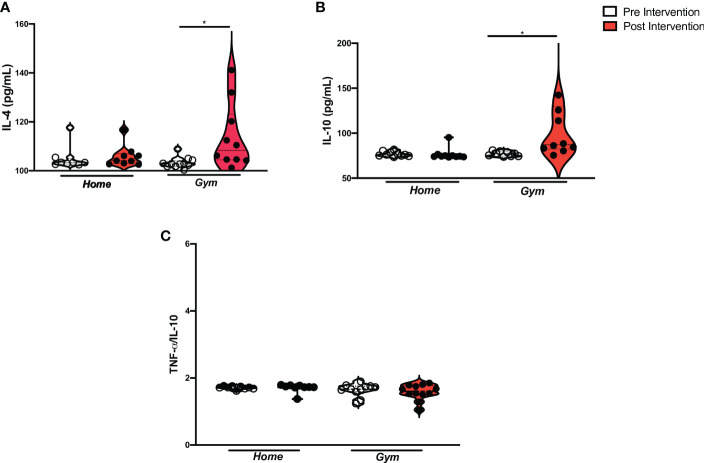
Anti-inflammatory cytokine levels in Supervised and home-based unsupervised exercise on recovered COVID-19 patients. ANOVA between Supervised and home-based unsupervised exercise on the anti-inflammatory cytokines and TNF-α/IL-10 ratio from baseline to 12 weeks of intervention (Pre- and Post-intervention). Home, the home-based unsupervised exercise group; Gym, supervised exercise group; Serum levels; **(A)** IL-4, Interleukin-4 (home (pre), n= 8, home (post), n= 12; gym (pre), n= 9, gym (post), n=11; *p* = 0.006); **(B)** IL-10: Interleukin-10 (home (pre), n= 10, home (post), n= 10; gym (pre), n= 10, gym (post), n=11; *p* = 0.04); **(C)** TNF-α/IL-10 ratio: (home (pre), n= 9, home (post), n= 9; gym (pre), n= 12, gym (post), n= 13; *p* = n.s.); pg/mL, picogram per milliliter; *p< 0.05.

We observed a statistically significant increase in the anti-inflammatory cytokines IL-4 (*p* = 0.006) and IL-10 (*p* = 0.04) serum levels in supervised exercise protocol compared to baseline (**Figure 4**). Furthermore, the IL-4 serum levels ranged non-significantly when the gym group was compared to the home group (Δ = 3.90 ± 21.37 pg/mL vs. Δ = -0.16 ± 3.26 pg/mL). The levels of IL-10 also did not change significantly in the gym group as compared to the home-based unsupervised group (Δ = 2.34 ± 31.08 pg/mL vs. Δ = -0.26 ± 6.49 pg/mL) (**Supplementary Figure 3**. On the other hand, we did not find a difference in the TNF-α: IL-10 ratio (*p* > 0.999) (**Figure 4**). Equally, we did not perceive changes in cytokine serum levels due to the home-based exercise after 12 weeks. Chemokine-cytokine-group comparisons are shown in **Figures 2** **–** **4**.

## Discussion

This study intended to investigate the effect of performing a supervised physical exercise (gym) on the serum levels of chemokines and cytokines compared to home-based unsupervised exercise, a strategy largely adopted in the COVID-19 pandemic due to the necessity of social isolation. Thus, our study provides the first evidence that demonstrates that supervised exercise could be required to mitigate the inflammatory effects in patients after COVID-19 recovery since this study has observed the modulation of serum levels of chemokines and cytokines by supervised exercise protocol.

Literature data show that during COVID-19 infection there is an increase in CCL-2/MCP-1 and CXCL8/IL-8 serum levels in response to cell recruitment, infiltration, and lung injury (54). Cytokines associated with lung injuries, such as IL-1 and TNF-α could induce the production of several chemokines, such as CCL2, CCL3, CCL5, and CXCL8, culminating in a cytokine-to-chemokine-to-cytokine signaling cascade through inflammatory response to the viral infection (55). Anderberg et al. (2021) have shown that IL-8 was responsible for neutrophil recruitment, activation, and storage, related to acute kidney injury as a consequence of severe COVID-19 (56). Studies have also shown high levels of IL-8 in different stages of COVID-19 recovery, associated with the inflammatory profile and peripheral neuronal disorders (28, 29). Further, we must highlight that CCL-2 is associated with macrophage recruitment. Hence, symptomatic (mild and severe forms) COVID-19 patients have high levels of CCL-2, together with patients dying from SARS-CoV-2 acute infection (57, 58). In this sense, Huang, C. et al. (2020) have observed that patients hospitalized due to their severe COVID-19 have high levels of CCL2/MCP-1 and CCL3 serum. These chemokines have been important biomarkers of severe disease, morbidity, and mortality (1). In this regard, it has been discussed that high production of some chemokines, such as CXCL8/IL-8, CCL-2/MCP-1, CCL3, CCL5, CXCL9, and CXCL10 are associated with lung damage and COVID-19 severity (59). However, the impact of high levels of most of these chemokines in the stages of COVID-19 recovery is unclear.

This is the first study that investigates the impact of supervised physical exercise and home-based unsupervised exercise addressing chemokines and cytokines in COVID-19 recovery. We observed a reduction of CCL-2/MCP-1 and CXCL8/IL-8 peripheral levels after 12 weeks of supervised exercise in patients post-COVID-19. Also, we have noted that while the CCL2 serum levels decreased in the supervised physical exercise group, they remained at altered levels in-home group despite six months of recovery (three first months before the intervention and 12 weeks of home-based unsupervised intervention). A study by Liu et al. (2020), investigating four weeks of two supervised exercise protocols (aerobic and strength training) in the elderly with dementia, has noted a decrease in CCL-2/MCP-1 serum levels in both supervised exercise protocols (60). In parallel, evidence about the IL-8 decrement was not conclusive in the chronic obstructive pulmonary disease (COPD) investigation, a chronic inflammatory lung disease, when it was attended due to supervised exercise (61), and also in home-based protocols for eight weeks of intervention (62, 63). One explanation for the divergent results may be the intervention time in both studies since we performed a 12-week protocol and Uzeloto et al. (2022) investigated eight weeks of supervised exercise protocols (61). Other studies evaluating a time course of physical exercise could better explain the kinetics of levels of these chemokines. In COVID-19, a significant reduction of the inflammatory process related to CCL-2/MCP-1 and CXCL8/IL-8 activity might be associated with the exercise protocol with controlled intensity and duration. For example, Cox et al. (2007) have observed that upper-respiratory disease-prone athletes have lower serum resting IL-8, IL-10 e IL-1ra concentrations, culminating in impaired inflammatory regulation (64).

To date, no published clinical trials have also investigated the effect of exercise protocols on cytokines in patients after COVID-19 recovery. This trial noticed that supervised exercise promoted a balance between pro-inflammatory and anti-inflammatory profiles highlighted by the increase in IL-2 and IL-6, as well as a rise in IL-4, IL-10, and a reduction in IFN-γ, IL-8, and CCL2. In contrast, home-based exercise was unsuccessful in modulating the inflammatory component. Associated with other results, it might indicate a remaining pro-inflammatory status in the home group that might be due to a deficient performance of the home-based exercise (less adhesion or not enough intensity). Additionally, we remarked on an enhancement in IL-2 serum levels in response to the supervised protocol. The increase in IL-2 secretion might be associated with T-cells activation. Moreover, IL-2 contributes to the Th1 response, mitigating infections caused by intracellular microorganisms (65, 66). In this sense, it is known that IL-2 might be an important biomarker for fighting against COVID-19 once T cells are antiviral players, but also mitigate the cytokine storm during the acute phase of COVID-19 (67). Therefore, a highlight in IL-2 appears to be crucial to immune surveillance against COVID-19.

Although the pleiotropic behavior of IL-6 is known, this study observed an increase in peripheral levels of this cytokine (68). Steensberg et al. (2003) have already observed an anti-inflammatory profile associated with IL-6 enhancement, such as a decrease in TNF-α and a marked increase in IL-1a and IL-10 plasma levels (36). The high IL-10 concentration counteracts the pro-inflammatory effects of some cytokines and cell infiltration by reducing the adhesion molecules (69, 70). Indeed, some studies have shown the production of myokines, such as IL-6, which are responsible for the modulatory effect of exercise, and exert anti-inflammatory outcomes (71). Moreover, experimental studies investigating influenza found an enhancement of mortality in IL-6 knockout mice because of a reduction in macrophage infiltration in the lung and epithelial cell survival, increased fibroblast proliferation, and collagen deposition (23, 72). On the other hand, other studies, which have investigated exercise protocols, have found a decrease in that cytokine in other viruses, and inflammatory diseases (73–76). These different IL-6 changes might be associated with different exercise protocols (frequency, intensity, and duration) (71). The higher levels of L-6, IL-4, and IL-10 which were observed in this study are associated with a cytokine profile of the post-COVID-19 population without sequelae as Queiroz et al. (2022) recently found out (7). Therefore, post-COVID-19 patients should also benefit from the anti-inflammatory effects of supervised exercise, which is still associated with increased IL-4, IL-10, and decreased chemokines levels.

Given the observation, this study supports some limitations. Although age was significantly different between groups, cytokine-chemokine levels showed no significant difference between supervised physical exercise and home-based unsupervised exercise groups. Moreover, the ANCOVA findings did not differ upon considering baseline age as a covariate for all chemokines and cytokines, except TNF-α levels in the gym group. The intensity of the home-based exercise protocol could not be measured. Also, this study could not consider a control group. However, our results are important since they were able to recognize the beneficial effect of supervised exercise in balancing pro-inflammatory and anti-inflammatory profiles after COVID-19 recovery. Future research should investigate the impact of long-term controlled exercise protocol (supervised and home-based) on patients after COVID-19 recovery. In addition, the most optimal conditions of a safe and effective exercise protocol remain to be determined (type, frequency, intensity, and duration).

## Conclusion

The current study indicated that a supervised controlled exercise protocol could balance the inflammatory peripheral components, mitigating the inflammatory process associated with post-COVID-19 and its consequences. Also, the supervised exercise might help to recover from COVID-19 disorders, enhancing immunosurveillance against this virus. Although the home-based unsupervised exercise was not able to decrease inflammation, future studies should not exclude the possibility of home-based with supervised exercise.

## Data availability statement

The original contributions presented in the study are included in the article/**Supplementary Material**. Further inquiries can be directed to the corresponding author.

## Ethics statement

The studies involving human participants were reviewed and approved by Research Ethics Board of The Federal University of Pernambuco. The patients/participants provided their written informed consent to participate in this study.

## Author contributions

PC conceived and designed the study approach. PC assisted in the exercise training of participants. TF, AC, and FS performed the immunological experiments. TF and MSSF executed statistical analysis. TF and MSSF wrote the manuscript. PC, AC, AT, JF, RBA, and FS contributed to critical revisions. All authors approved the final manuscript.

## Supplementary material

Supplementary Figure 1 Changes in chemokines from baseline to 12 weeks of Supervised and home-based unsupervised exercise on recovered COVID-19 patients. Comparison of variations between Supervised and home-based unsupervised exercise on the chemokines’ serum levels according to T-test from baseline to 12 weeks of intervention (delta of Post- to Pre-intervention). Home, the home-based unsupervised exercise group; Gym, supervised exercise group; Serum levels; **(A)** IL-8: Interleukin- 8 (*p* = 0.74); **(B)** CCL5: CC chemokine family-5 (*p* = 0.06); **(C)** CXCL10: chemokine (C-X-C motif) ligand-10 (*p* = 0.04); D) CXCL9: chemokine (C-X-C motif) ligand-9 (*p* = 0.13); E) CCL2: CC chemokine family-2 (*p* = 0.11); pg/mL: picogram per milliliter; *p< 0.05.

Supplementary Figure 2 Changes in pro-inflammatory cytokines from baseline to 12 weeks of Supervised and home-based unsupervised exercise on recovered COVID-19 patients. Comparison of variations between Supervised and home-based unsupervised exercise on the pro-inflammatory cytokines’ serum levels according to T-test from baseline to 12 weeks of intervention (delta of Post- to Pre-intervention). Home, the home-based unsupervised exercise group; Gym, supervised exercise group; Serum levels; **(A)** IL-2: Interleukin-2 (*p* = 0.04); **(B)** IL-6: Interleukin-6 (*p* = 0.15); **(C)** TNF-α: Tumor necrosis factor-alpha (*p* = 0.46; **(D)** IFN-γ: Interferon-gamma (*p* = 0.95); E) IL-17A: Interleukin-17A (*p* = 0.01); pg/mL: picogram per milliliter; *p< 0.05.

Supplementary Figure 3 Changes in anti-inflammatory cytokines from baseline to 12 weeks of Supervised and home-based unsupervised exercise on recovered COVID-19 patients. Comparison of variations between Supervised and home-based unsupervised exercise on the anti-inflammatory cytokines’ serum levels according to T-test from baseline to 12 weeks of intervention (delta of Post- to Pre-intervention). Home, the home-based unsupervised exercise group; Gym, supervised exercise group; Serum levels; **(A)** IL-4: Interleukin-4 (*p* = 0.14); **(B)** IL-10: Interleukin-10 (*p* = 0.88); **(C)** TNF-α/IL-10 ratio: (*p* = 0.60); pg/mL: picogram per milliliter; *p< 0.05.
