# Supplementary material for: The impact of supervised physical exercise on chemokines and cytokines in recovered COVID-19 patients
Source: Front Immunol. 2023 Jan 4;13:1051059. doi: 10.3389/fimmu.2022.1051059 (PMC9846636; doi:10.3389/fimmu.2022.1051059)
Supplement: Supplementary file 1 [file Table_1.docx]

| **Items** |  | **Home** |  | **Gym** | **Kruskal-Wallis** | **ANCOVA** |
| --- | --- | --- | --- | --- | --- | --- |
|  | Pre-intervention | Post-intervention | Pre-intervention | Post-intervention | *p* | *p* |
| **Chemokines** |  |  |  |  |  |  |
| IL-8 (pg/mL) | 218.2 (163.5-350.0) | 182.5 (159.9-286.9) | 272.8 (157.7-545.3) | 161.3 (153.7-354.6) ***** | **0.04** | 0.06 |
| CCL2 (pg/mL) | 1124 (384.9-2773) | 992.1 (557.3-2997) | 808.9 (423.9-1123) | 445.9 (263.9-710.2) ***** | **0.03** | 0.73 |
| CCL5 (pg/mL) | 112666 (89361-121063) | 107284 (100856-116095) | 118521 (100027-130180) | 117927 (112985-119972) | *>0.99* | 0.57 |
| CXCL9 (pg/mL) | 1101 (548.0-4518) | 1484 (566.6-6296) | 531.9 (307.7-915.1) | 666.4 (213.5-1298) | *>0.99* | 0.13 |
| CXCL10 (pg/mL) | 5846 (1322-27128) | 1709 (1085-17778) | 1503 (565.7-.5427) | 1198 (556.8-5390) | *>0.99* | 0.49 |
| **Cytokines** |  |  |  |  |  |  |
| IL-2 (pg/mL) | 35.43 (34.87-38.84) | 35.40 (34.65-36.06) | 36.07 (35.29-37.38) | 37.95 (35.23-108.7) ***** | **0.02** | 0.53 |
| IL-4 (pg/mL) | 103.4 (102.5-117.6) | 104.2 (102.5-116.8) | 102.8 (99.22-108.9) | 108.3 (101.3-141.2) ***** | **0.006** | 0.54 |
| IL-6 (pg/mL) | 113.1 (107.4-186.1) | 107.7 (102.7-116.6) | 111.2 (106.5-169.4) | 154.0 (120.9-461.7) ***** | **0.03** | 0.58 |
| IL-10 (pg/mL) | 218.2 (163.5-350.0) | 182.5 (159.9-286.9) | 272.8 (157.7-545.3) | 161.3 (153.7-354.6) ***** | **0.04** | 0.50 |
| IL-17A (pg/mL) | 94.22 (81.51-128.3) | 92.77 (84.55.-94.71) | 93.11 (87.18-128.7) | 116.8 (87.73-261.8) | 0.79 | 0.68 |
| IFN-γ (pg/mL) | 36.34 (34.06-81.83) | 36.40 (34.90-38.75) | 38.19 (35.10-51.71) | 34.83 (33.07-36.48) ***** | **0.004** | 0.35 |
| TNF-α (pg/mL) | 130.4 (126.1-132.5) | 130.6 (127.5-132.2) | 130.2 (128.3-149.9) | 132.0 (126.2-146.9) | *>0.99* | **0.00^#^** |
| TNF-α /IL-10 (pg/mL) | 1.716 (1.617-1.765) | 1.731 (1.373-1.796) | 1.690 (1.240-3.221) | 1.542 (0.273-1.850) | *>0.99* | 0.98 |

**Caption:** IL: Interleukin, CXCL: chemokine (C-X-C motif) ligand, CCL: CC chemokine family, TNF-α: Tumor necrosis factor-alpha, IFN-y: Interferon-gamma, *Significance between the gym pre- vs post-intervention using the Kruskal–Wallis test with multiple comparisons and the Dunn post-test; # Significance in the post-intervention the gym group controlling for the age as a covariate by one-way analysis of covariance (ANCOVA). Values are median (Min-Max).

**Supplementary Table 1.** Impacts of home-based unsupervised exercise and supervised exercise on chemokines and cytokines from baseline to 12 weeks of intervention.
