# Supplementary figures and images for: The impact of supervised physical exercise on chemokines and cytokines in recovered COVID-19 patients
Source: Front Immunol. 2023 Jan 4;13:1051059. doi: 10.3389/fimmu.2022.1051059 (PMC9846636; doi:10.3389/fimmu.2022.1051059)

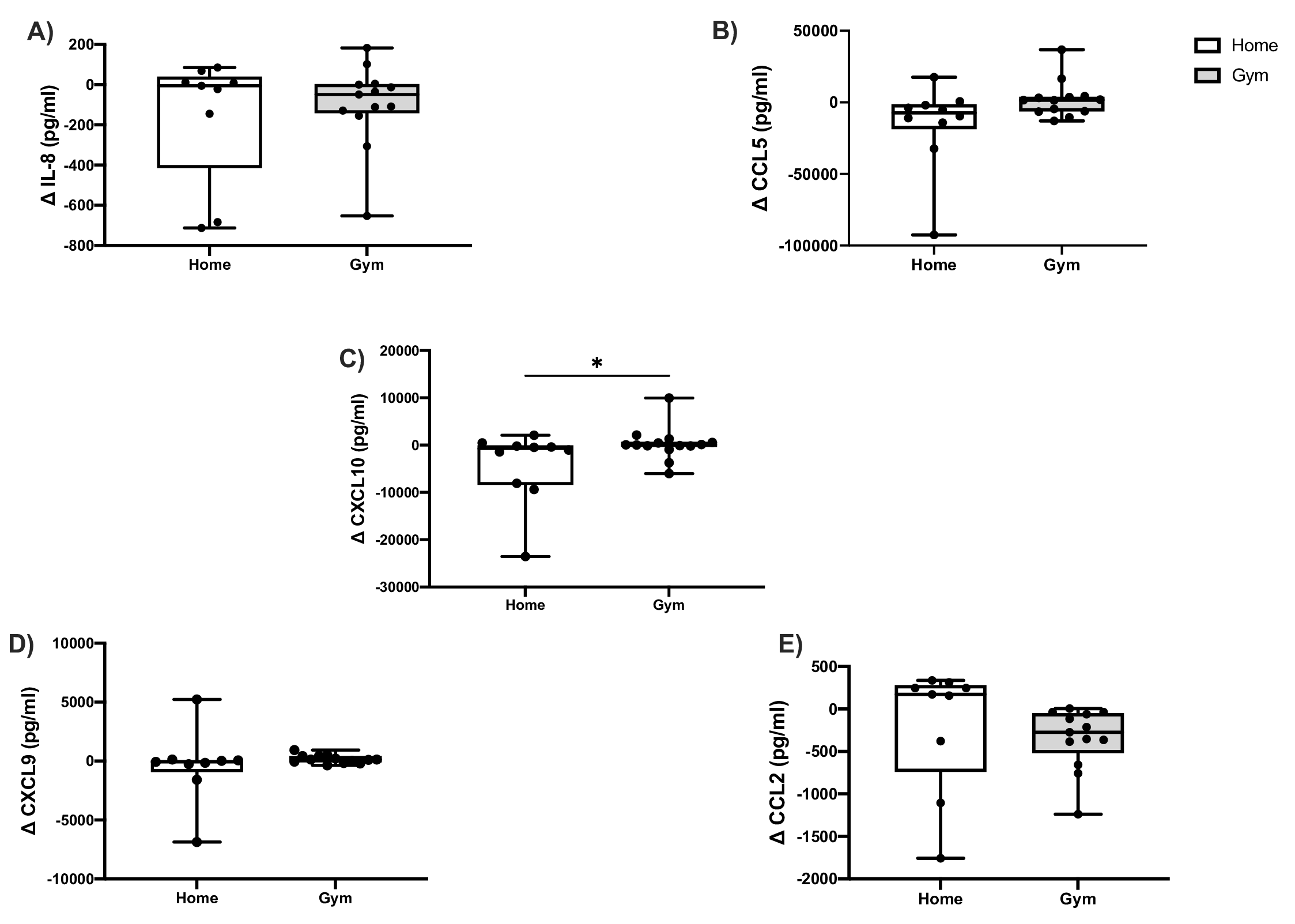

Supplement: Supplementary Figure 1 — Changes in chemokines from baseline to 12 weeks of Supervised and home-based unsupervised exercise on recovered COVID-19 patients. Comparison of variations between Supervised and home-based unsupervised exercise on the chemokines’ serum levels according to T-test from baseline to 12 weeks of intervention (delta of Post- to Pre-intervention). Home, the home-based unsupervised exercise group; Gym, supervised exercise group; Serum levels; (A) IL-8: Interleukin- 8 (p = 0.74); (B) CCL5: CC chemokine family-5 (p = 0.06); (C) CXCL10: chemokine (C-X-C motif) ligand-10 (p = 0.04); D) CXCL9: chemokine (C-X-C motif) ligand-9 (p = 0.13); E) CCL2: CC chemokine family-2 (p = 0.11); pg/mL: picogram per milliliter; *p< 0.05. [file Image_1.tiff]

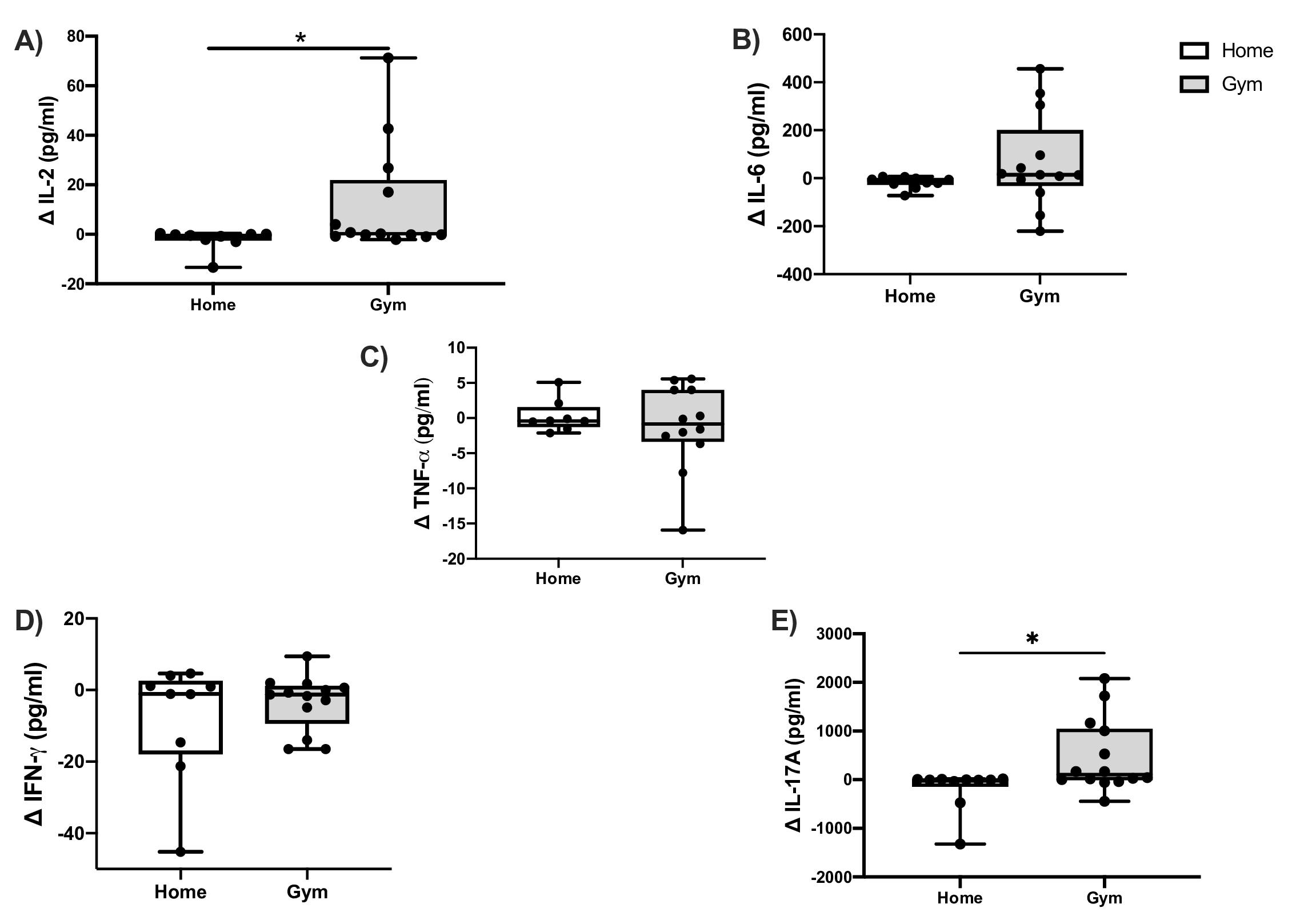

Supplement: Supplementary Figure 2 — Changes in pro-inflammatory cytokines from baseline to 12 weeks of Supervised and home-based unsupervised exercise on recovered COVID-19 patients. Comparison of variations between Supervised and home-based unsupervised exercise on the pro-inflammatory cytokines’ serum levels according to T-test from baseline to 12 weeks of intervention (delta of Post- to Pre-intervention). Home, the home-based unsupervised exercise group; Gym, supervised exercise group; Serum levels; (A) IL-2: Interleukin-2 (p = 0.04); (B) IL-6: Interleukin-6 (p = 0.15); (C) TNF-α: Tumor necrosis factor-alpha (p = 0.46; (D) IFN-γ: Interferon-gamma (p = 0.95); E) IL-17A: Interleukin-17A (p = 0.01); pg/mL: picogram per milliliter; *p< 0.05. [file Image_2.tiff]

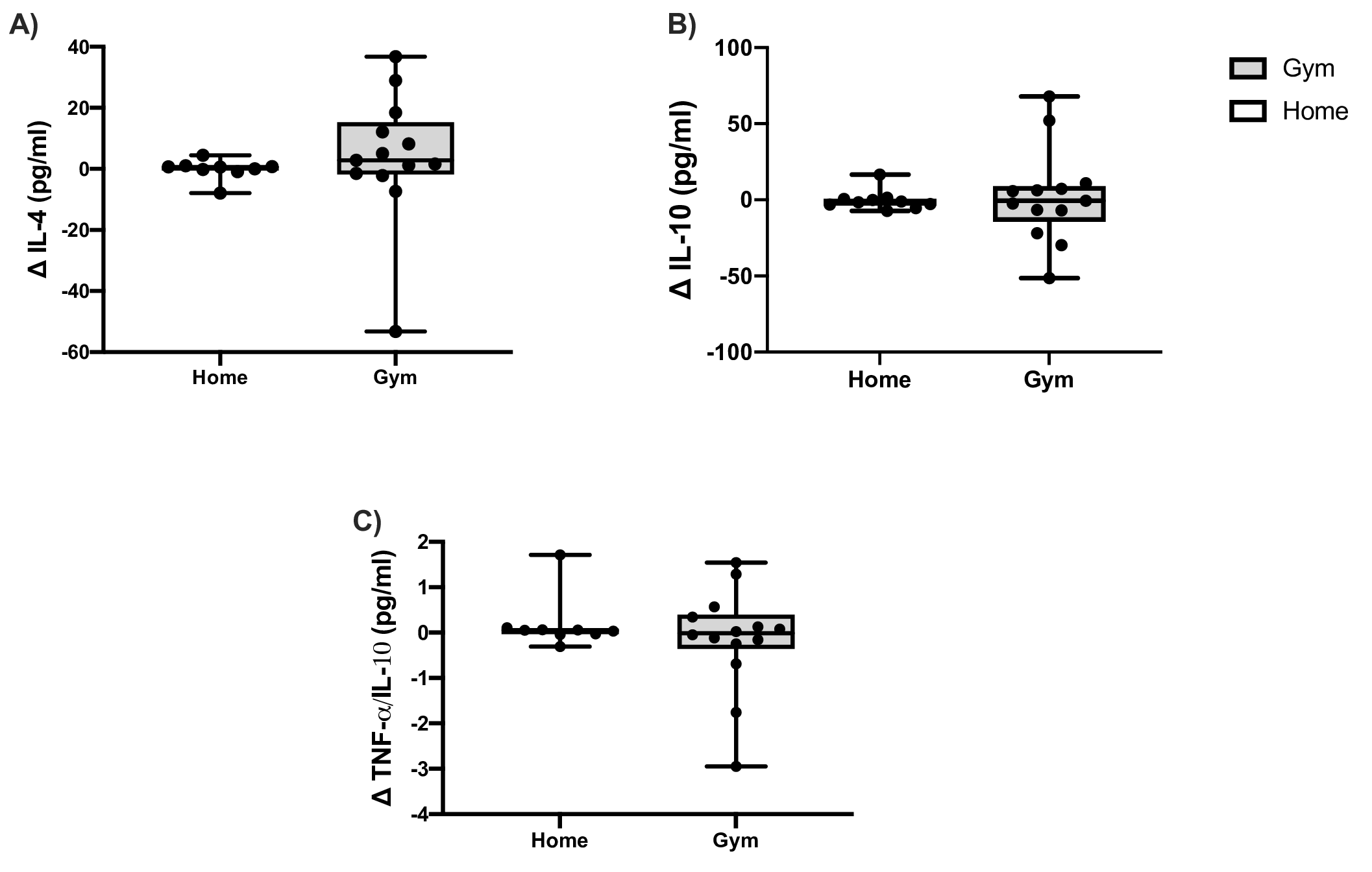

Supplement: Supplementary Figure 3 — Changes in anti-inflammatory cytokines from baseline to 12 weeks of Supervised and home-based unsupervised exercise on recovered COVID-19 patients. Comparison of variations between Supervised and home-based unsupervised exercise on the anti-inflammatory cytokines’ serum levels according to T-test from baseline to 12 weeks of intervention (delta of Post- to Pre-intervention). Home, the home-based unsupervised exercise group; Gym, supervised exercise group; Serum levels; (A) IL-4: Interleukin-4 (p = 0.14); (B) IL-10: Interleukin-10 (p = 0.88); (C) TNF-α/IL-10 ratio: (p = 0.60); pg/mL: picogram per milliliter; *p< 0.05. [file Image_3.tiff]
